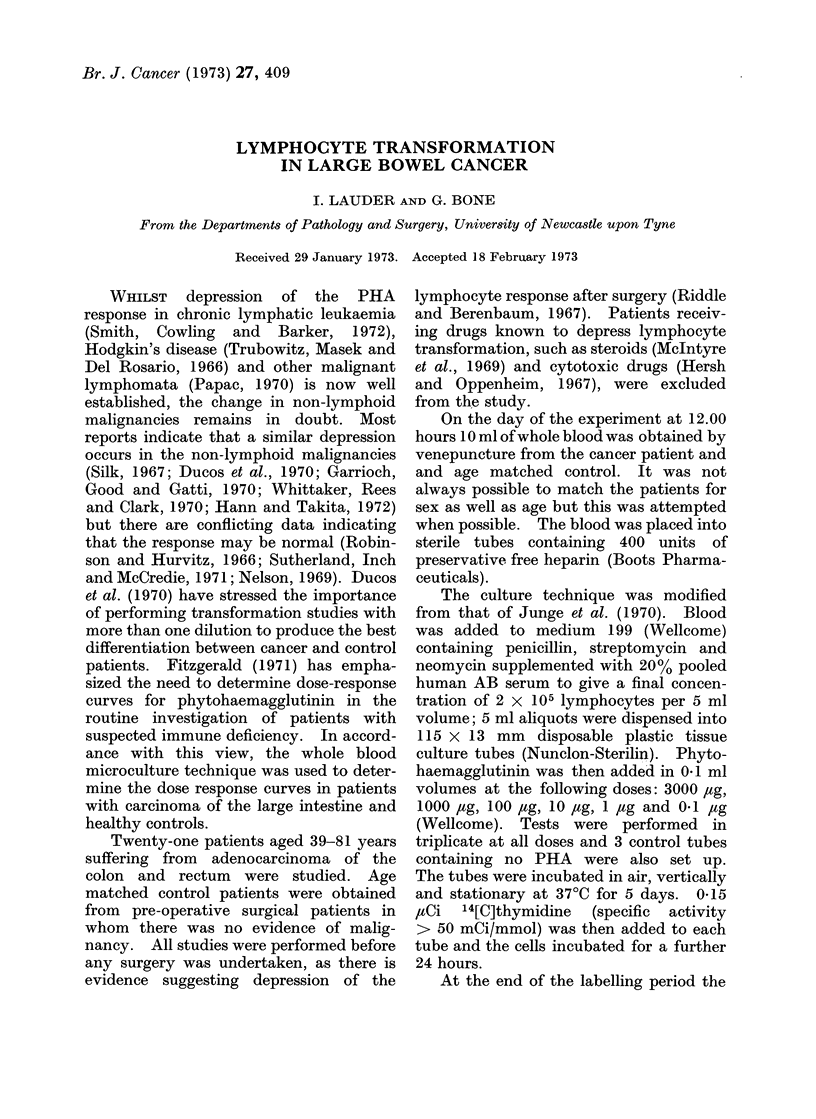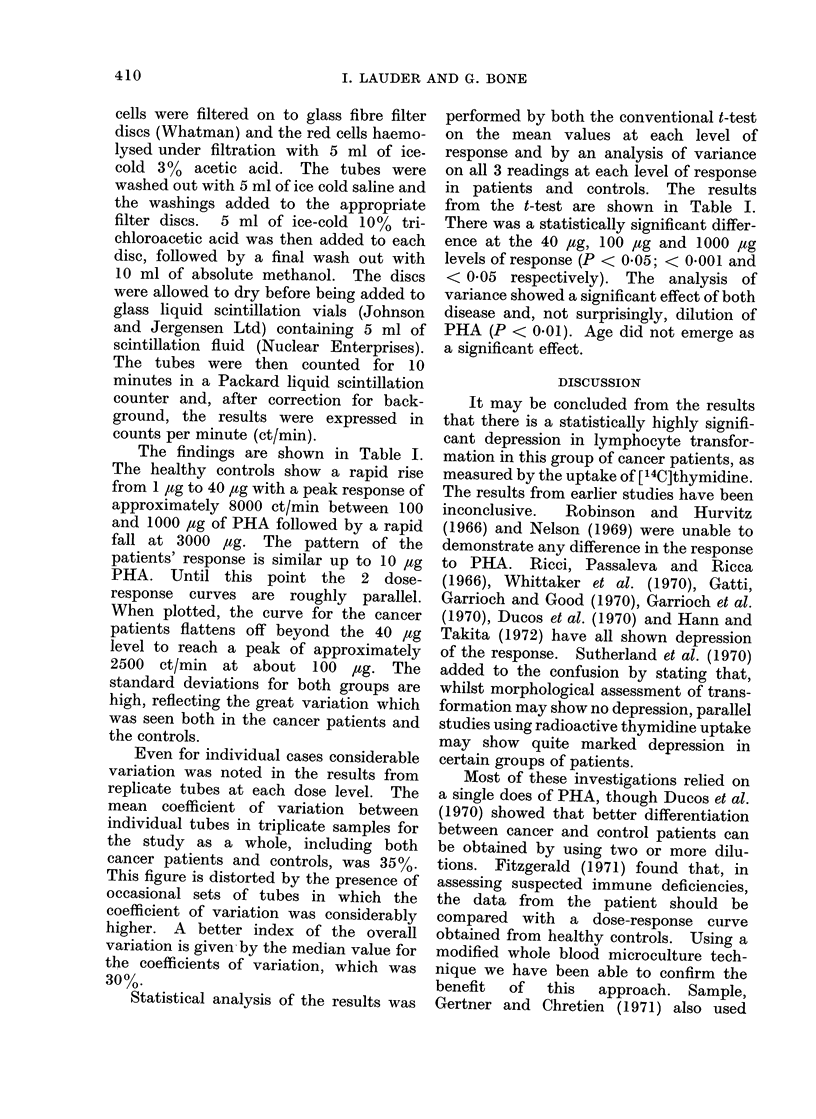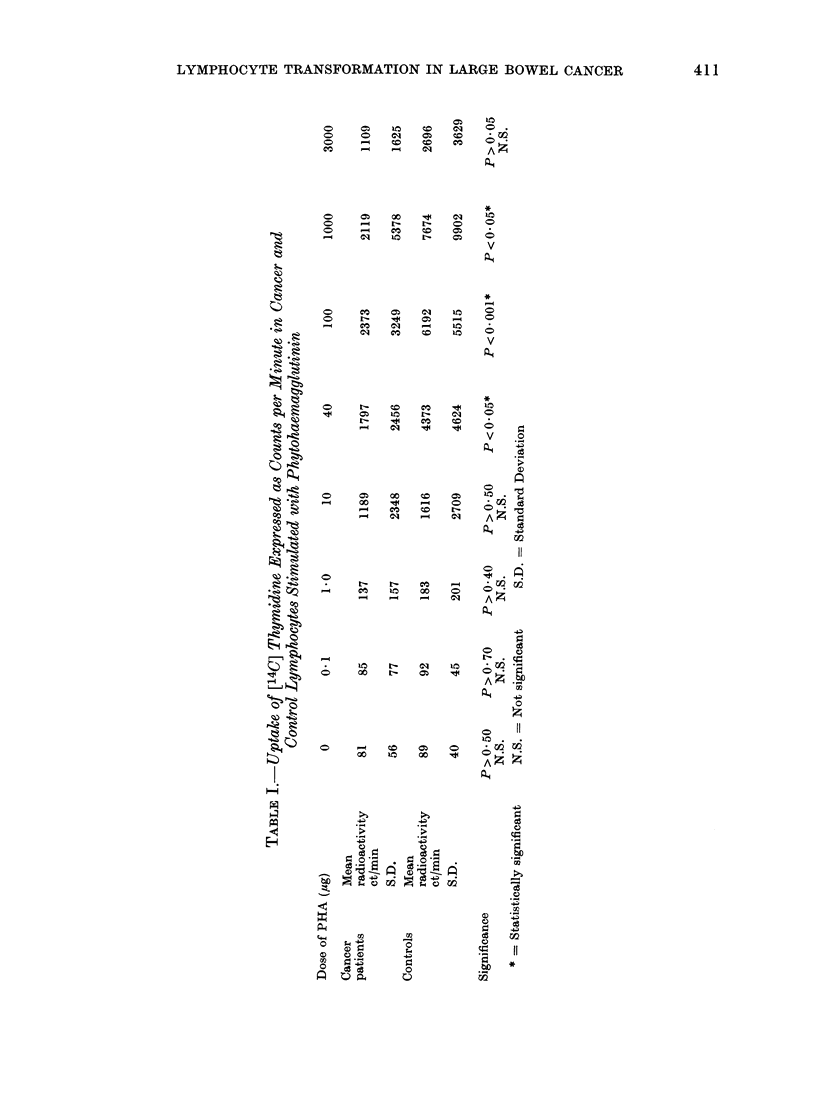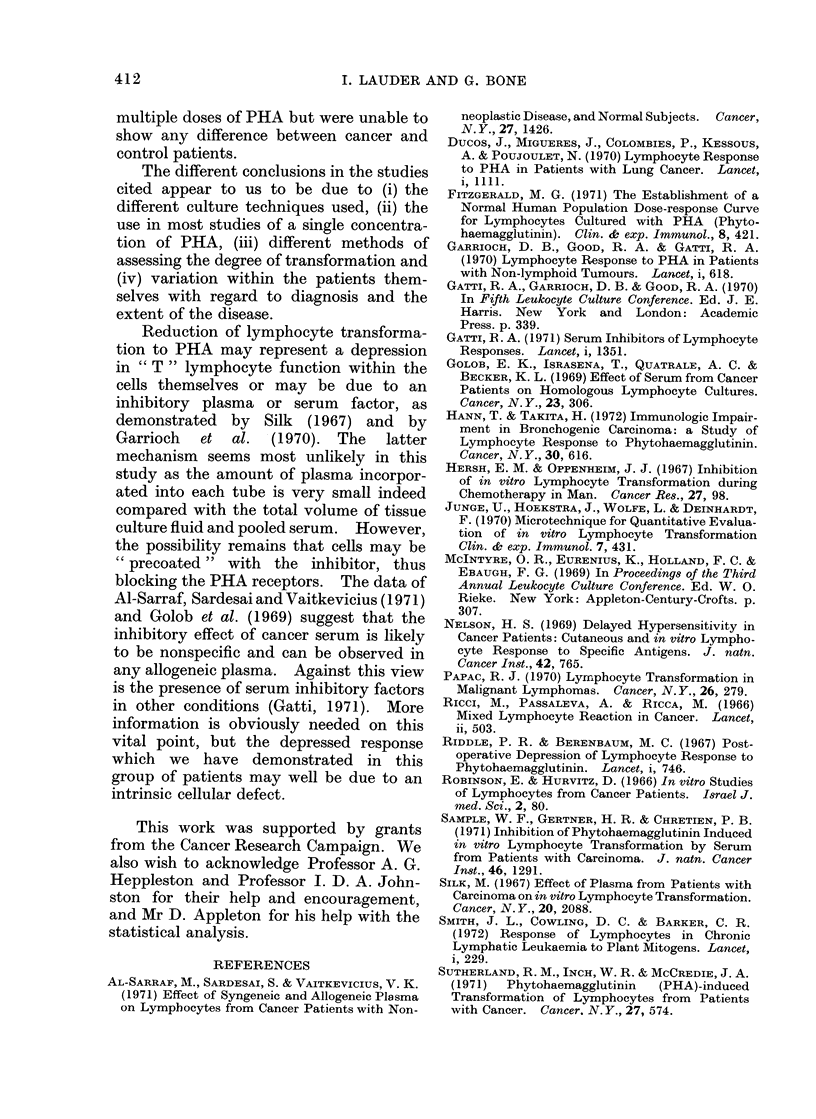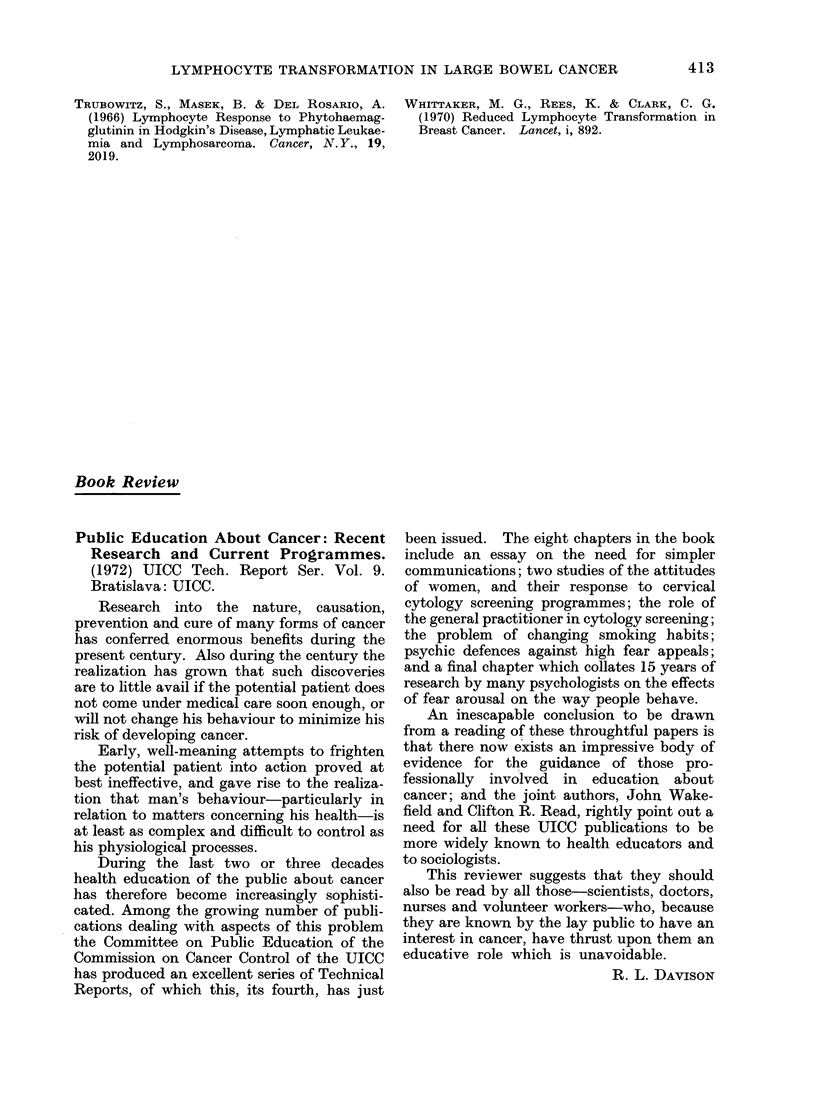# Lymphocyte transformation in large bowel cancer.

**DOI:** 10.1038/bjc.1973.50

**Published:** 1973-05

**Authors:** I. Lauder, G. Bone


*Br. J. Cancer* (1973) **27**, 409

### LYMPHOCYTE TRANSFORMATION IN LARGE BOWEL CANCER

I. LAUDER AND G. BONE

*From the Departments of Pathology and Surgery, University of Newcastle upon Tyne*

Received 29 January 1973. Accepted 18 February 1973

WHILST depression of the PHA response in chronic lymphatic leukaemia (Smith, Cowling and Barker, 1972), Hodgkin's disease (Trubowitz, Masek and Del Rosario, 1966) and other malignant lymphomata (Papac, 1970) is now well established, the change in non-lymphoid malignancies remains in doubt. Most reports indicate that a similar depression occurs in the non-lymphoid malignancies (Silk, 1967; Ducos *et al.*, 1970; Garrioch, Good and Gatti, 1970; Whittaker, Rees and Clark, 1970; Hann and Takita, 1972) but there are conflicting data indicating that the response may be normal (Robinson and Hurvitz, 1966; Sutherland, Inch and McCredie, 1971; Nelson, 1969). Ducos *et al.* (1970) have stressed the importance of performing transformation studies with more than one dilution to produce the best differentiation between cancer and control patients. Fitzgerald (1971) has emphasized the need to determine dose-response curves for phytohaemagglutinin in the routine investigation of patients with suspected immune deficiency. In accordance with this view, the whole blood microculture technique was used to determine the dose response curves in patients with carcinoma of the large intestine and healthy controls.

Twenty-one patients aged 39–81 years suffering from adenocarcinoma of the colon and rectum were studied. Age matched control patients were obtained from pre-operative surgical patients in whom there was no evidence of malignancy. All studies were performed before any surgery was undertaken, as there is evidence suggesting depression of the lymphocyte response after surgery (Riddle and Berenbaum, 1967). Patients receiving drugs known to depress lymphocyte transformation, such as steroids (McIntyre *et al.*, 1969) and cytotoxic drugs (Hersh and Oppenheim, 1967), were excluded from the study.

On the day of the experiment at 12.00 hours 10 ml of whole blood was obtained by venepuncture from the cancer patient and and age matched control. It was not always possible to match the patients for sex as well as age but this was attempted when possible. The blood was placed into sterile tubes containing 400 units of preservative free heparin (Boots Pharmaceuticals).

The culture technique was modified from that of Junge *et al.* (1970). Blood was added to medium 199 (Wellcome) containing penicillin, streptomycin and neomycin supplemented with 20% pooled human AB serum to give a final concentration of $2 \times 10^5$ lymphocytes per 5 ml volume; 5 ml aliquots were dispensed into $115 \times 13$ mm disposable plastic tissue culture tubes (Nunclon-Sterilin). Phytohaemagglutinin was then added in 0·1 ml volumes at the following doses: 3000 $\mu$g, 1000 $\mu$g, 100 $\mu$g, 10 $\mu$g, 1 $\mu$g and 0·1 $\mu$g (Wellcome). Tests were performed in triplicate at all doses and 3 control tubes containing no PHA were also set up. The tubes were incubated in air, vertically and stationary at 37°C for 5 days. 0·15 $\mu$Ci $^{14}$[C]thymidine (specific activity $> 50$ mCi/mmol) was then added to each tube and the cells incubated for a further 24 hours.

At the end of the labelling period the cells were filtered on to glass fibre filter discs (Whatman) and the red cells haemolysed under filtration with 5 ml of ice-cold 3% acetic acid. The tubes were washed out with 5 ml of ice cold saline and the washings added to the appropriate filter discs. 5 ml of ice-cold 10% trichloroacetic acid was then added to each disc, followed by a final wash out with 10 ml of absolute methanol. The discs were allowed to dry before being added to glass liquid scintillation vials (Johnson and Jergensen Ltd) containing 5 ml of scintillation fluid (Nuclear Enterprises). The tubes were then counted for 10 minutes in a Packard liquid scintillation counter and, after correction for background, the results were expressed in counts per minute (ct/min).

The findings are shown in Table I. The healthy controls show a rapid rise from 1 μg to 40 μg with a peak response of approximately 8000 ct/min between 100 and 1000 μg of PHA followed by a rapid fall at 3000 μg. The pattern of the patients' response is similar up to 10 μg PHA. Until this point the 2 dose-response curves are roughly parallel. When plotted, the curve for the cancer patients flattens off beyond the 40 μg level to reach a peak of approximately 2500 ct/min at about 100 μg. The standard deviations for both groups are high, reflecting the great variation which was seen both in the cancer patients and the controls.

Even for individual cases considerable variation was noted in the results from replicate tubes at each dose level. The mean coefficient of variation between individual tubes in triplicate samples for the study as a whole, including both cancer patients and controls, was 35%. This figure is distorted by the presence of occasional sets of tubes in which the coefficient of variation was considerably higher. A better index of the overall variation is given by the median value for the coefficients of variation, which was 30%.

Statistical analysis of the results was performed by both the conventional *t*-test on the mean values at each level of response and by an analysis of variance on all 3 readings at each level of response in patients and controls. The results from the *t*-test are shown in Table I. There was a statistically significant difference at the 40 μg, 100 μg and 1000 μg levels of response ($P < 0{\cdot}05$; $< 0{\cdot}001$ and $< 0{\cdot}05$ respectively). The analysis of variance showed a significant effect of both disease and, not surprisingly, dilution of PHA ($P < 0{\cdot}01$). Age did not emerge as a significant effect.

### DISCUSSION

It may be concluded from the results that there is a statistically highly significant depression in lymphocyte transformation in this group of cancer patients, as measured by the uptake of [$^{14}$C]thymidine. The results from earlier studies have been inconclusive. Robinson and Hurvitz (1966) and Nelson (1969) were unable to demonstrate any difference in the response to PHA. Ricci, Passaleva and Ricca (1966), Whittaker *et al.* (1970), Gatti, Garrioch and Good (1970), Garrioch *et al.* (1970), Ducos *et al.* (1970) and Hann and Takita (1972) have all shown depression of the response. Sutherland *et al.* (1970) added to the confusion by stating that, whilst morphological assessment of transformation may show no depression, parallel studies using radioactive thymidine uptake may show quite marked depression in certain groups of patients.

Most of these investigations relied on a single does of PHA, though Ducos *et al.* (1970) showed that better differentiation between cancer and control patients can be obtained by using two or more dilutions. Fitzgerald (1971) found that, in assessing suspected immune deficiencies, the data from the patient should be compared with a dose-response curve obtained from healthy controls. Using a modified whole blood microculture technique we have been able to confirm the benefit of this approach. Sample, Gertner and Chretien (1971) also used multiple doses of PHA but were unable to show any difference between cancer and control patients.

The different conclusions in the studies cited appear to us to be due to (i) the different culture techniques used, (ii) the use in most studies of a single concentration of PHA, (iii) different methods of assessing the degree of transformation and (iv) variation within the patients themselves with regard to diagnosis and the extent of the disease.

Reduction of lymphocyte transformation to PHA may represent a depression in " T " lymphocyte function within the cells themselves or may be due to an inhibitory plasma or serum factor, as demonstrated by Silk (1967) and by Garrioch *et al.* (1970). The latter mechanism seems most unlikely in this study as the amount of plasma incorporated into each tube is very small indeed compared with the total volume of tissue culture fluid and pooled serum. However, the possibility remains that cells may be " precoated " with the inhibitor, thus blocking the PHA receptors. The data of Al-Sarraf, Sardesai and Vaitkevicius (1971) and Golob *et al.* (1969) suggest that the inhibitory effect of cancer serum is likely to be nonspecific and can be observed in any allogeneic plasma. Against this view is the presence of serum inhibitory factors in other conditions (Gatti, 1971). More information is obviously needed on this vital point, but the depressed response which we have demonstrated in this group of patients may well be due to an intrinsic cellular defect.

This work was supported by grants from the Cancer Research Campaign. We also wish to acknowledge Professor A. G. Heppleston and Professor I. D. A. Johnston for their help and encouragement, and Mr D. Appleton for his help with the statistical analysis.

### REFERENCES

AL-SARRAF, M., SARDESAI, S. & VAITKEVICIUS, V. K. (1971) Effect of Syngeneic and Allogeneic Plasma on Lymphocytes from Cancer Patients with Non-neoplastic Disease, and Normal Subjects. *Cancer, N.Y.*, **27**, 1426.

DUCOS, J., MIGUERES, J., COLOMBIES, P., KESSOUS, A. & POUJOULET, N. (1970) Lymphocyte Response to PHA in Patients with Lung Cancer. *Lancet*, i, 1111.

FITZGERALD, M. G. (1971) The Establishment of a Normal Human Population Dose-response Curve for Lymphocytes Cultured with PHA (Phytohaemagglutinin). *Clin. & exp. Immunol.*, **8**, 421.

GARRIOCH, D. B., GOOD, R. A. & GATTI, R. A. (1970) Lymphocyte Response to PHA in Patients with Non-lymphoid Tumours. *Lancet*, i, 618.

GATTI, R. A., GARRIOCH, D. B. & GOOD, R. A. (1970) In *Fifth Leukocyte Culture Conference*. Ed. J. E. Harris. New York and London: Academic Press. p. 339.

GATTI, R. A. (1971) Serum Inhibitors of Lymphocyte Responses. *Lancet*, i, 1351.

GOLOB, E. K., ISRASENA, T., QUATRALE, A. C. & BECKER, K. L. (1969) Effect of Serum from Cancer Patients on Homologous Lymphocyte Cultures. *Cancer, N.Y.*, **23**, 306.

HANN, T. & TAKITA, H. (1972) Immunologic Impairment in Bronchogenic Carcinoma: a Study of Lymphocyte Response to Phytohaemagglutinin. *Cancer, N.Y.*, **30**, 616.

HERSH, E. M. & OPPENHEIM, J. J. (1967) Inhibition of *in vitro* Lymphocyte Transformation during Chemotherapy in Man. *Cancer Res.*, **27**, 98.

JUNGE, U., HOEKSTRA, J., WOLFE, L. & DEINHARDT, F. (1970) Microtechnique for Quantitative Evaluation of *in vitro* Lymphocyte Transformation *Clin. & exp. Immunol.* **7**, 431.

McINTYRE, O. R., EURENIUS, K., HOLLAND, F. C. & EBAUGH, F. G. (1969) In *Proceedings of the Third Annual Leukocyte Culture Conference*. Ed. W. O. Rieke. New York: Appleton-Century-Crofts. p. 307.

NELSON, H. S. (1969) Delayed Hypersensitivity in Cancer Patients: Cutaneous and *in vitro* Lymphocyte Response to Specific Antigens. *J. natn. Cancer Inst.*, **42**, 765.

PAPAC, R. J. (1970) Lymphocyte Transformation in Malignant Lymphomas. *Cancer, N.Y.*, **26**, 279.

RICCI, M., PASSALEVA, A. & RICCA, M. (1966) Mixed Lymphocyte Reaction in Cancer. *Lancet*, ii, 503.

RIDDLE, P. R. & BERENBAUM, M. C. (1967) Postoperative Depression of Lymphocyte Response to Phytohaemagglutinin. *Lancet*, i, 746.

ROBINSON, E. & HURVITZ, D. (1966) *In vitro* Studies of Lymphocytes from Cancer Patients. *Israel J. med. Sci.*, **2**, 80.

SAMPLE, W. F., GERTNER, H. R. & CHRETIEN, P. B. (1971) Inhibition of Phytohaemagglutinin Induced *in vitro* Lymphocyte Transformation by Serum from Patients with Carcinoma. *J. natn. Cancer Inst.*, **46**, 1291.

SILK, M. (1967) Effect of Plasma from Patients with Carcinoma on *in vitro* Lymphocyte Transformation. *Cancer, N.Y.*, **20**, 2088.

SMITH, J. L., COWLING, D. C. & BARKER, C. R. (1972) Response of Lymphocytes in Chronic Lymphatic Leukaemia to Plant Mitogens. *Lancet*, i, 229.

SUTHERLAND, R. M., INCH, W. R. & McCREDIE, J. A. (1971) Phytohaemagglutinin (PHA)-induced Transformation of Lymphocytes from Patients with Cancer. *Cancer, N.Y.*, **27**, 574.

TRUBOWITZ, S., MASEK, B. & DEL ROSARIO, A. (1966) Lymphocyte Response to Phytohaemagglutinin in Hodgkin's Disease, Lymphatic Leukaemia and Lymphosarcoma. *Cancer, N.Y.*, **19**, 2019.

WHITTAKER, M. G., REES, K. & CLARK, C. G. (1970) Reduced Lymphocyte Transformation in Breast Cancer. *Lancet*, i, 892.